# Lhermitte’s Sign——A Neuropathic Pain and Its Neuroanatomy

**DOI:** 10.21315/mjms2024.31.5.21

**Published:** 2024-10-08

**Authors:** Pei Ting Heng, Zaitun Zakaria, Diana Noma Fitzrol, Muhammad Ihfaz Ismail, Song Yee Ang, Nelson Kok Bing Yap, Hui Mei Tan

**Affiliations:** 1 Department of Neurosciences, School of Medical Sciences, Universiti Sains Malaysia, Kelantan, Malaysia; 2 Brain and Behaviour Cluster, School of Medical Sciences, Universiti Sains Malaysia, Kelantan, Malaysia; 3 Department of Neurosciences and Brain Behaviour Cluster, Hospital Universiti Sains Malaysia, Universiti Sains Malaysia, Kelantan, Malaysia; 4 Department of Neurosurgery, Sibu Hospital, Sarawak, Malaysia; 5 Department of Anaesthesiology, Sarawak General Hospital, Sarawak, Malaysia

Dear Editor,

We read with interest an article by Nallaluthan et al. (1). We would like to mention about the Lhermitte’s pain sign and explain its neuroanatomy origin pathway interpretation.

Lhermitte’s sign, also known as Lhermitte’s phenomenon or barber chair phenomenon was first described in 1917 by Pierre Marie and Jean-Charles Chatelin and named after Jean Lhermitte (2). It is a type of neuropathic pain characterised by a transient sensation of electric shock from the head down the spine and occasionaly through the arms and legs when the neck is flexed (i.e. when their head is bent down) or simply when they touch their chin to their chest (2, 3). In one case, it was reported to have been triggered by fatigue, stress, heat and yawning (2).

Limited statistics have been reported on the incidence or prevalence of Lhermitte’s sign. Its aetiologies have been reported as multiple sclerosis (MS), spinal cord compression or myelopathy, radiculopathy, syringomyelia, trauma, vitamin B12 deficiency, subacute combined degeneration of the cord, chemoradiation and nitric oxide toxicity (2, 3). However, most epidemiological studies have referred to it in MS patients (3). A prospective study in 2015 reported low sensitivity of MS patients to this condition, with only an approximately 16% incidence (4). However, an earlier study published in 1994 reported its 97% specificity in patients with compressive myelopathy (5). This could indicate that Lhermitte’s sign has been an underestimated red flag in clinical practice.

Lhermitte’s sign affects the dorsal column, involving both extra-axial and intra-axial lesions. When the neck is flexed, the mechanoreceptors in the cervical region, especially those along the posterior aspect, undergo stretching. These mechanoreceptors include those in ligaments, muscles, tendons, facet joints, intervertebral discs and meninges (Table 1). This stretching is particularly intense at the C4/C5 level, which is why Lhermitte’s sign indicates an upper cervical cord lesion (6).

The stretching of the cord, particularly at its posterior aspect involving the dorsal column, dorsal horn, and dorsal root entry zone, triggers an impulse that is sent to Rexed laminae V, where the mechanical nociceptors are located (6). This signal then ascends via the lateral spinothalamic tract to reach the ventral posterolateral nuclei in the thalamus, where it is relayed to the primary somatosensory cortex (6).

The efferent pathway involves the primary somatosensory cortex sending signals down to the periaqueductal gray and then to the rostral ventromedial medulla. The impaired function of inhibitory GABAergic interneurons due to pro-inflammatory cytokine signalling, which is a result of the disease process, augments the pain (7).

Lhermitte’s sign is a complex condition with various proposed mechanisms. One paper mentioned that patient described Lhermitte’s sign as non-painful paraesthesia which was elucidated as ‘electric feeling’ in all fingers of both hands and occasionally in anterior parts of thighs (8).

Traditionally, it is attributed to three main mechanisms (8, 9). In MS cases, it often arises from the hyperexcitability of demyelinated neurons (9)—that is, to the increased responsiveness of these neurons to various sensory inputs. Abnormal nerve firing can occur not only in neuroma but also demyelination plaques (8). These injured nerves are more likely to fire repetitively and spontaneously (8). Conversely, Lhermitte’s sign can be caused by compression or irritation of neurons at the dorsal root in conditions such as cervical spondylosis. These injured axons tend to be mechanosensitive and, thus, more prone than normal axons to the excitability induced by mechanical stimulation (8–10). This explains the tingling sensation induced by physical movement due to the physical distortion or stretching of injured axons, which leads to ectopic firing of nerves. Additionally, ephaptic transmission between physically adjacent fibres is thought to play a role in Lhermitte’s sign (9, 11). An ephapse refers to a site of lateral contact between juxtaposed demyelinated axons through which impulses are conducted directly across the nerve membranes (9, 11).

Several other theories have been proposed to explain the pathophysiology of neuropathic pain. These theories highlight the multifaceted nature of neuropathic pain and underscore the need for comprehensive approaches to its management. The ascending and descending pathway are illustrated in Figure 1 and Figure 2.

## Pathophysiology of Neuropathic Pain

### Pain Signalling Change

Chronic neuropathic pain is characterised by a complex interplay of physiological and molecular changes that alter the normal processing of pain signals. One key aspect involves the alteration of the electrical properties of sensory nerves (12, 13). These changes disrupt the balance between excitatory and inhibitory signalling within the central nervous system (CNS), impair the inhibitory interneurons and descending control systems that normally help regulate pain signals (13) and lead to a state of hyperexcitability in the sensory pathways, where pain signals are amplified and transmitted more easily (14).

Over time, these changes can contribute to the development of chronic neuropathic pain. The sequence of events that leads to chronicity is thought to involve a cascade of changes that occur from the periphery to the brain. These changes may include sensitisation of peripheral nerves, alterations in spinal cord processing and changes in the brain’s response to pain signals (7, 10). This process is complex and multifaceted, involving both peripheral and central mechanisms, and contributes to the persistence of pain even after the initial injury or damage has healed (11, 13).

## Adaptation in Ion Channels

Changes in ion channels—specifically in sodium, calcium and potassium channels—within affected nerves play a critical role in the amplification and maintenance of pain signals (9, 13). One of the more important of these changes is the increased expression and function of sodium channels, particularly at the spinal cord terminus of sensory nerves (13). This heightened sodium channel activity increases neuronal excitability, enhances the signal transmission and increases the release of neurotransmitters, all of which contribute to the heightened perception of pain.

The upregulation of the α2δ subunit of calcium channels further enhances the neuronal excitability and neurotransmitter release (13). On the other hand, potassium channels that normally help regulate neural activity are reduced, leading to further disruptions in signalling processes (13).

In addition, when an afferent fibre is severed from the periphery due to an injury or lesion, sensory loss typically occurs in the affected area. However, the residual fibres at the injury site can exhibit ectopic activity, such as spontaneous firing, which can lead to a sensation of pain in areas that would otherwise have reduced sensation or would be numb (10).

Moreover, the fibres that remain in the affected area become hyperexcitable and turn into irritable nociceptors (13). This heightened excitability can result in the generation of spontaneous pain signals and increased sensitivity to stimuli, contributing to the perception of pain from stimuli that would otherwise be normal (known as allodynia) and to the persistence and aggravation of pain (known as hyperalgesia).

## Second Order Neuron Adjustment

Changes in second-order nociceptive neurons are pivotal in the development and persistence of chronic neuropathic pain. These changes lead to an increased responsiveness of these neurons to various sensory inputs (8–10), which allows for the activation of these neurons by low-threshold mechanosensitive Aβ and Aδ afferent fibres, which typically do not elicit pain signals (10, 13). Thus, the receptive fields of these neurons expand, which means that a broader population of them can now be activated by a stimulus—a process referred to as central sensitisation (7).

These adaptations are driven by ongoing activity in peripheral afferent fibres, which release excitatory amino acids and neuropeptides. This leads to postsynaptic changes in second-order nociceptive neurons, including an excessive signalling cascade due to the phosphorylation of N-methyl-D-aspartic acid (NMDA) and α-amino-33-hydroxy-5-methyl-4-isoxazolepropionic acid (AMPA) receptors (7, 13). This excess signalling is believed to contribute to physical allodynia, where non-painful stimuli provoke pain sensations. The hyperexcitability of second-order nociceptive neurons is further evidenced by increased activity in ventrolateral and ventromedial nuclei of thalamus.

Moreover, the loss of GABA-releasing inhibitory interneurons, which normally dampen neuronal activity, can also contribute to this hyperexcitability (13). In neuropathic pain state, these interneurons can switch to exert excitatory actions, exacerbating the hyperexcitable state of second-order nociceptive neurons.

## Changes In Inhibitory Modulation

Patients experiencing neuropathic pain often present with dysfunctional inhibitory interneurons and descending modulatory control systems. Brain regions such as the cingulate cortex and amygdala are implicated in maintaining ongoing pain states and associated comorbidities. These forebrain areas project to the periaqueductal grey, a key centre for descending pain modulation, which in turn acts on spinal pain signalling (13).

In neuropathic pain, there is a reduction in noradrenergic inhibitions mediated by α2-adrenergic receptors in the spinal cord, while there is an increase in serotonin signalling through the 5-HT2 and 5-HT3 receptors (13). This shift in neurotransmitter balance contributes to the dominance of enhanced serotonin signalling in neuropathic pain states. The noradrenergic system is crucial for mediating diffuse noxious inhibitory controls (DNIC), which are often impaired or lost in neuropathic pain conditions (13).

## Pain Modulation Mechanism

Neuropathic pain affects patients to varying degrees, with some experiencing moderate discomfort while others endure debilitating pain. A key factor contributing to this variability is the modulation of the pain message within the CNS (7, 13). The pain signal can be either amplified or attenuated as it travels from its point of entry, the dorsal horn, through the CNS to reach the cerebral cortex.

In neuropathic pain, many patients exhibit a pro-nociceptive pain modulation profile, indicating that pain messages are amplified within the CNS (7, 13). This amplification can lead to a heightened perception of pain due to reduced descending endogenous inhibition (13). This inhibition is often reflected in less-efficient conditioned pain modulation (CPM) (13).

Additionally, sensitisation of ascending pain pathways can further enhance the perception of pain, as indicated by increased temporal summation of painful stimuli. A rodent study has shown that pro-inflammatory cytokines such as TNF-α, IFN-ψ, IL-1⎬, IL-6, IL-12 and IL-18 plays a role in central sensitisation through its their disruption on the integrity of blood brain barrier (7). The interplay between these mechanisms can lead to the disinhibition of pain perception in patients with neuropathic pain.

A good example of this pain modulation mechanism is depicted by the role of melatonin receptors in modulation of low back pain secondary to intervertebral disc degeneration. Melatonin not only regulates neuropathic pain but also has potent antioxidant, anti-inflammatory and anti-apoptotic effect (15). It has been reported that in the initial phase of intervertebral disc degeneration, the expression of melatonin receptor MT2 is upregulated by nucleus pulposus in intervertebral disc as an adaptive response (15). As the disease progresses and low back pain persists, it stimulates the expression of melatonin receptor MT1 (15). This stimulation of MT1 aids in pain modulation, providing analgesic effect on low back pain (15). It also inhibits apoptosis in nucleus pulposus (15). Studies have shown significant reduction of reported low back pain in patients who have upregulated MT1 and MT2 expressions (15).

## Diagnosis

There is no gold standard tests, be it method, biomarker, neurophysiological test or imaging to diagnose neuropathic pain (3). To make matter more arduous, some patients not only have neuropathic pain but also nociceptive pain (2, 3, 13). There are currently several self-reported questionnaire for neuropathic pain such as Douleur Neuropathique en 4 Questions, Neuropathic Pain Questionnaire and painDETECT questionnaire (13). Another validated screening tool that is commonly used in clinical trials due to its contribution to quantifying neuropathic symptoms and phenotyping is the Neuropathic Pain Symptom Inventory (13). A paper published in 2016 proposed an upgraded system for grading neuropathic pain into: possible, probable and definite (16). The pain is deemed possible if the patient’s history suggests a neurological lesion with neuroanatomically plausible pain distribution (16); probable if such pain distribution is confirmed from clinical examination (16); and definite if it is positively diagnosed in a test (16).

Ancillary tests that are available and routinely used are quantitative sensory tests and neurophysiological assessments (13). Quantitative sensory tests use mechanical and thermal stimuli to assess afferent nociceptive and non-nociceptive stimuli in peripheral and CNS (13). This is possible as different afferent fibres are affected by respective stimuli. Compared to nerve conduction studies and the somatosensory evoked potential test, the most reliable neurophysiological test is the laser-evoked potential test, which provides information on pain pathways (13).

## Treatment

Management of neuropathic pain is impeccably tricky. The most practised approach is initiate pharmacological treatment prior to interventional therapies. The pharmacological treatment is usually aimed at relieving symptoms by targeting ion channels and second-order neurons, as well as at modulating the inhibitory pathway. The complexity of neuropathic pain mechanisms induces various treatment responses. However, psychological factors also play a role.

### First-Line Treatment

Antidepressants and antiepileptics show promising efficacy in the first-line treatment of neuropathic pain. Tricyclic antidepressants inhibit monoamine reuptake and sodium channels, whereas serotonin/noradrenaline reuptake inhibitors block serotonin and noradrenaline reuptake in the descending inhibitory pathway (13, 17, 18). Antiepileptics such as pregabalin and gabapentin reduce central sensitisation by binding to the α_2_δ subunit of voltage-gated calcium channels (13, 17, 18). When monotherapy is refractory, combination therapy is routinely applied; and combination therapy in moderate doses is justified when high-dose monotherapy is intolerable (17).

### Second-Line Treatment

Tramadol is a synthetic opioid which acts as centrally acting μ-receptor agonist and serotonin/noradrenaline reuptake inhibitor (13, 17). Its efficacy is well established in peripheral neuropathic pain but less promising in central neuropathic pain (13). Topical patches such as Lidocaine and Capsaicin are useful especially in postherpetic neuralgia (13, 17). Lidocaine inhibits sodium channel and thus blocking ectopic neuronal discharge (13, 17, 18). On the other hand, Capsaicin desensitises transient receptor potential cation channel subfamily V member 1 (TRPV1) (13, 17, 18).

### Third-Line Treatment

Botulinium toxin A inhibits acetylcholine release and poses potential effects on mechanotransduction in peripheral neuropathic pain (13). Its efficacy improves with enhanced effect of repeated administration (13). Other treatment options include opioid (other than Tramadol) and antiepileptics (other than a28 subunit of voltage-gated calcium channel agonist) may be considered but their efficacies are inconsistent (13).

### Interventional Therapies

Interventional treatment encompasses neural blockade, surgeries to deliver drugs to targeted areas or eradicate causative lesion and neuromodulation. Neural blockade usually involves combined steroid and local anaesthetic injection. Its efficacy is well established in pain relief and functional outcome. Sympathetic ganglion block seems promising but the modality requires more studies to evaluation its efficacy.

Neuromodulation techniques such as dorsal root ganglion neurostimulation, epidural motor cortex stimulation (ECMS), repetitive transcranial magnetic stimulation (rTMS) and transcranial direct current stimulation (tDCS) of the precentral motor cortex are effective in pain reduction in patients with chronic neuropathic pain (13, 18). ECMS is an invasive neurosurgical procedure in which stimulating electrode is placed over motor cortex that corresponds to the homunculus, whereas rTMS and tDCS are non-invasive procedures in which magnetic coils or electrodes are placed over the scalp (13, 18). The recommended regime for rTMS is 5–10 sessions of 5 Hz–20 Hz of rTMS over 1 week–2 weeks (13). Although rTMS and tDCS are non-invasive, they are contraindicated in patients with a history of epilepsy; an intracranial implant, such as a deep brain electrode, cochlear implant or aneurysm clip; or a cardiac implant, such as a pacemaker or a defibrillator (13).

Ablative procedures, such as dorsal root entry zone lesionectomy and thalamotomy, are effective alternatives (18). However, despite their promising results, their clinical use has been reduced over the years due to their irreversibility (18). Thalamotomy is applied to stop thalamocortical dysrhythmia, which has been hypothesised as the culprit in neuropathic pain, as it induces ectopic neuronal discharges between the thalamus and the cortical regions (18). Central lateral thalamotomy is aimed at the posterior aspect of the central lateral nucleus and, thus, modulates thalamocortical activity, which then helps to alleviate pain (18).

## Conclusion

Managing neuropathic pain is challenging due to its multifaceted nature, which makes its pathophysiology intricate. Thus, understanding the pathophysiology mechanisms of neuropathic pain is crucial for its effective treatment.

## Figures and Tables

**Figure 1:**
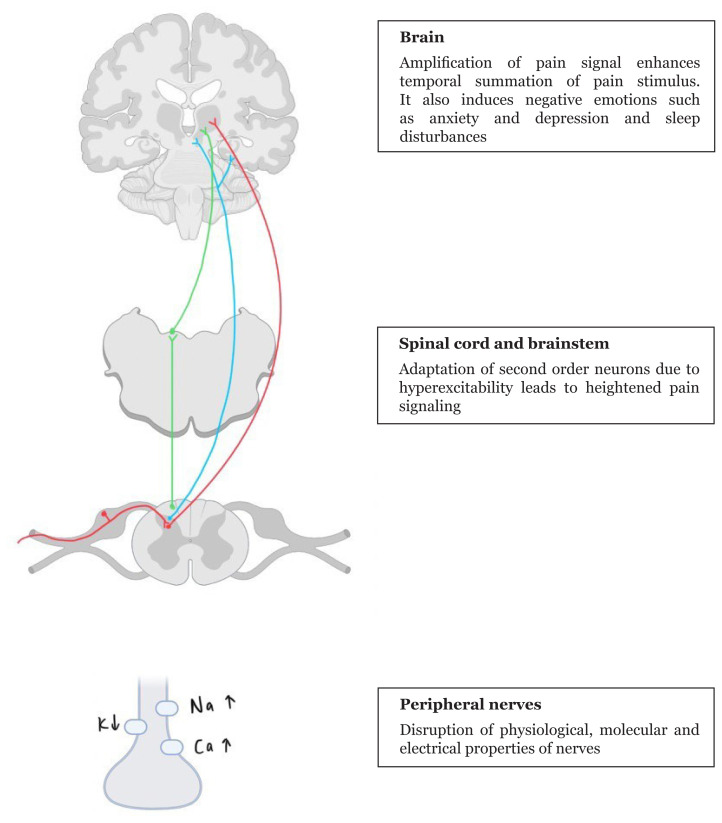
A summary of ascending pain pathway. Damage to sensory nerve fibres (Aβ, Aδ and C afferent fibres) leads to changes in regulation of ion channels. These changes alter the electrical properties, which then affects the transduction and transmission of pain signal. The adaptation of second order neurons carries the heightened pain signals to brain, where thalamus relays the pain messages to somatosensory cortex. The red line depicts the spinothalamic tract; the light blue line, limbic pathway; and the green line, ascending reticular activating system including dorsal column pathway

**Figure 2:**
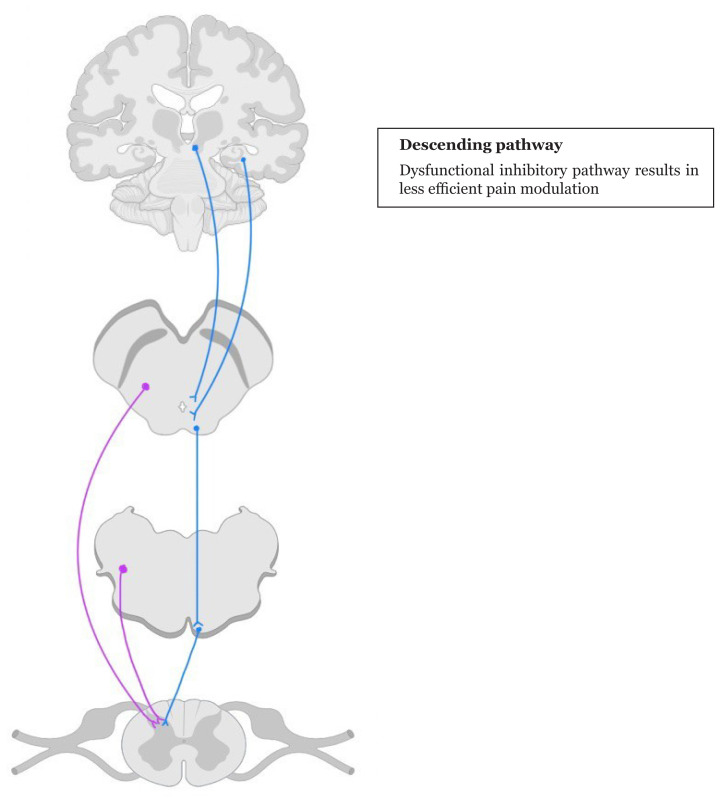
A summary of descending pain pathway. Loss of noradrenergic inhibition and enhancement of serotonergic pathway impair the balance of neurotransmitters that are essential for pain modulation. The purple line depicts the noradrenaline pathway and the blue line, the descending inhibitory pathway

**Table 1:** Summary of mechanoreceptors

Structure	Mechanoreceptors
Ligament	Ruffini corpuscles, Pacinian corpuscles
Tendon	Golgi tendon bodies
Muscles	Extrafusal and intrafusal fibres
Facet joint	Ruffini corpuscles, Pacinian corpuscles, Golgi tendon bodies
Intervertebral disc	Ruffini corpuscles, Sino-vertebral nerve endings
Meninges	Various, e.g. calcitonin gene-related peptide receptors
